# Bio-Based Formulations for Sustainable Applications in Agri-Food-Pharma

**DOI:** 10.3390/biom11050768

**Published:** 2021-05-20

**Authors:** Minaxi Sharma, Rajeev Bhat, Zeba Usmani, David Julian McClements, Pratyoosh Shukla, Vinay B. Raghavendra, Vijai Kumar Gupta

**Affiliations:** 1 Food (By-) Products Valorisation Technologies (VALORTECH) ERA Chair, Estonian University of Life Sciences, 51006 Tartu, Estonia or minaxi.sharma@emu.ee (M.S.); or rajeev.bhat@emu.ee (R.B.); 2 Department of Applied Biology, University of Science and Technology, Meghalaya 793101, India; zeba24@gmail.com; 3 Department of Food Science, University of Massachusetts, Amherst, MA 01003, USA; McClements@foodsci.UMASS.edu; 4 Institute of Science, School of Biotechnology, Banaras Hindu University, Varanasi 221005, India; pratyoosh.shukla@gmail.com; 5 Enzyme Technology and Protein Bioinformatics Laboratory, Department of Microbiology, Maharshi Dayanand University, Rohtak 124001, Haryana, India; 6 P.G. Department of Biotechnology, Teresian College, Siddarthanagar, Mysore 570011, India; viragh79@gmail.com; 7 Biorefining and Advanced Materials Research Center, Scotland’s Rural College (SRUC), Edinburgh EH9 3JG, UK; 8 Center for Safe and Improved Food, Scotland’s Rural College (SRUC), Edinburgh EH9 3JG, UK

Currently, there is a strong enduring interest towards obtaining high-value, sustainable bio-based bioactive compounds from natural resources, as there is great demand for these compounds in various market sectors such as agriculture, food, pharma, cosmeceuticals, and others. This demand has encouraged researchers to isolate, identify and characterize novel natural bioactive compounds with potential therapeutic and commercial values with industrial importance [1]. These bioactive compounds are generally secondary metabolites (synthesized via plant biosynthetic pathways) and include polyphenols, carotenoids, flavonoids, sterols, dietary fiber, essential vitamins, coenzyme Q, phytosterols, glucosinolates and others with potential beneficial roles as nutraceuticals, surfactants and bio-stimulants. Understanding the molecular characteristics, physicochemical properties, biological activity, and stability of these bioactives under different conditions is vital for their commercial exploitation. The efficacy of these bioactives can often be improved by encapsulating them in nanobased-formulations designed for application in the agriculture, food, pharmaceutical industries. These delivery systems can be designed to increase the dispersibility, stability, bioavailability, and bioactivity imparted by the bioactives. Moreover, they may be useful for minimizing undesirable side-effects, facilitating targeted delivery to certain cells, and enhancing the shelf life of food products.

The bioactive molecules are partly or wholly derived from resources of biological origin mainly those of plants, animal and microbial resources (e.g., biomass/feed stock from agri-food sector, food wastes and by-products, algae, marine organisms, etc.). These molecules have recently emerged on the global market as a highly reliable environmentally friendly alternative to chemically synthesized compounds. The natural bioactive compounds provide additional benefits to health and overall wellbeing beyond basic nutrition. For instance, bioactive compounds have been well established for their antioxidant, antimicrobial, antiviral, anticancer, anti-hypertensive and other biological activities under in vitro and in vivo conditions. The isolation, purification and safety efficacy of these compounds obtained from natural resources is a vital criterion that needs to be considered [2].

This Special Issue, “Bioactive Formulations in Agri-Food-Pharma: Source and Applications”, has aimed to gather knowledge on recent advancements in the formulation of nano-delivery systems of novel bioactive compounds using advanced nano-engineered strategies. Additionally, it highlights the novel nano-formulations strategies that have been developed to enhance the bioactive potential of biobased compounds in food, agriculture, and pharmaceutical applications.

This Special Issue comprises of 13 research articles on bioactive compounds from plants, animals, and microbial sources that exemplify their bio-techno-functional properties to play a beneficial role in agri-food-pharma sector with valuable applications to benefit human health.

Further, a Web of Science search with keywords such as ‘Antimicrobial nanoformulations’, ‘Antioxidants nanoformulations’, ‘Drug nanoformulations’, ‘Anticancer nanoformulations’, ‘Plant nanoformulations’, ‘Essential oil nanoformulations’, ‘Pharmaceutical nanoformulations’, ‘Food nanoformulations’, ‘Nutraceutical nanoformulations’, ‘Therapeutic nanoformulations’ was carried out. Accordingly, the publication trends revealed a rapid growth in the scientific interest in these areas (see Figure 1). This search highlighted enhanced interest in the development of novel nanoformulation strategies for many kinds of bioactive agents. Much of this work, focused on the development of nanoparticle-based delivery systems to improve the stability, bioavailability, and efficacy of the bioactive compounds.

As per the information available, several researchers have documented their work on the potentiality of biobased bioactive compounds obtained from plant, animal or microbial resources, who have developed the nanoformulations by using different encapsulation strategies for the effective delivery of these compounds at targeted sites. Some case studies have been discussed in the below text.

*Alchornea cordifolia*, a popular African medicinal plant that is generally used for various medicinal applications, mainly for its antimicrobial activity against various species of bacteria, fungi, and parasites, as well as its possible efficacy against inflammatory disorders. The article by Sinan et al. [3] has focused on the total phenolic and flavonoid contents of infusion extracts of *A. cordifolia* leaves and have assessed its chemical composition. The findings of the study described the bioactive potential of leaf extracts such as enzyme inhibition and cytotoxicity activities on HepG2: human hepatocellular carcinoma cells, B16 4A5: murine melanoma cells, and S17: murine bone marrow (normal) cells. Bio-pharmacological effects of the extracts were ascertained and the potential mechanisms of action of the bioactive compounds in this medicinal plant were investigated using docking analysis. The researchers have reported on the presence of a number of bioactive compounds, including gallic acid, ellagic acid, shikimic acid, rutin, quercetin, myricetin, vitexin, quercitrin, kaempferol, and naringenin in methanolic and infusion extracts. Additionally, these extracts showed good cytotoxic activity against human hepatocellular carcinoma cells. This study demonstrated that *A. cordifolia* could be a suitable plant source to further investigate the mechanism of action of the bioactive compounds present (as antioxidant and anticancer agents) for potential pharmaceutical applications.

On the other hand, Chaudhary et al. [4] produced natural polymers with enhanced moisture absorption/retention properties, which were proved to aid in the conditioning of soil and improvement in the soil health, specifically in water deficient areas. The authors formulated agar–agar (Agr) and gelatin (GE) copolymerized methyl acrylate (MA) and acrylic acid (AA) hydrogels (Agr/GE-co-MA/AA) by utilizing microwave-assisted green synthesis. By using Agr/GE-co-MA/AA hydrogels as moisture retaining agents, the water holding time was prolonged from 10 to 30 days for soil and 6 to 10 days for sand. This study showed that novel moisture retaining agents for soil, which are biodegradable, non-toxic, and of low-cost may be developed and applied in areas where water scarcity occurs.

Chowdappa et al. [5] isolated bioactive siderophores from endophytic fungi obtained from *Cymbidium aloifolium* (medicinal orchid plant) and studied their potential application as antimicrobial agents against phytopathogens in crop plants. These antimicrobial siderophores acted as effective chelating agents with transition metals, forming soluble complexes with Fe^3+^. The authors also showed that these siderophores inhibited virulent plant pathogens such as *Ralstonia solanacearum* (responsible for bacterial wilt in groundnut) and *Xanthomonas oryzae* pv. *oryzae* (responsible for bacterial blight in rice). The authors reported that *Penicillium chrysogenum* to be the best among all of the tested fungi and may be utilized in the form of bioactive formulations for development of resistance against other phytopathogens in crop plants.

Eco-friendly metal nanoparticles can be produced by certain bacterial isolates that have potent antibacterial and antifungal activity. Mondal et al. [6] synthesized silver nanoparticles (AgNPs) extracellularly using an environmental bacterial isolate *Citrobacter* spp. MS5 culture supernatant. These biosynthesized AgNPs showed effective antibacterial activity against extended spectrum β-lactamase (ESBL) producing multidrug resistant Gram-negative bacteria. The AgNPs were reported to exhibit high antifungal activity towards pathogenic *Candida* spp.

Sudarsan et al. [7] optimized an eco-friendly approach to synthesize AgNPs using endophytic bacteria (*Cytobacillus firmus*) isolated from the stem bark of *Terminalia arjuna*. These silver nanoparticles effectively inhibited bacterial growth in a dose-dependent manner. Results of this study showed good antimicrobial and antifungal activities against Gram-positive (*Staphylococcus aureus)* and Gram-negative (*Escherichia coli)* bacteria, as well as pearl millet blast disease-causing fungi (*Magnoporthe grisea*). They also described the role of endophytic secondary metabolites in metal reduction, stabilization, and capping of silver nanoparticles.

On the other note, nanotechnology applications are being used to convert antioxidant and antimicrobial rich essential oils into water-dispersible forms, thereby enhancing their efficacy. Hemp is a widely cultivated crop to produce fibers and nutrients. The ability of hemp essential oil as act as an antimicrobial, anti-leishmanial and antioxidant agent has been reported recently by Menghini et al. [8]. The essential oils of three different cultivars of hemp (*Futura 75*, *Carmagnola selezionata* and *Eletta campana*), were evaluated for their antiprotozoal efficacy by administering them to mice intraperitoneally, which had initially been infected with *Leishmania tropica*. The bio-pharmacological activity was exhibited by the compound selina-3,7(11)-diene, which revealed its affinity in the micromolar range towards proliferator-activated receptor α, cannabinoid CB2 receptor and acetylcholinesterase. *Futura 75* and *E. campana* cultivars showed higher content of selina-3,7(11)-diene, and had higher scavenging/reducing properties with stronger efficacy against the tissue wound induced by *L. tropica*.

Nanotechnology has been used to create topical delivery systems for enhancing the utilization and efficacy of bioactivities. A combinatorial approach was reported to develop nanoemulsion-based delivery systems containing imiquimod in combination with curcumin to improve the topical delivery of these bioactive compounds [9]. In the co-delivery system, curcumin acts as a therapeutic agent that minimizes the adverse skin reactions that are frequently related with the topical therapy of imiquimod. The nanoemulsion was dispersed in a hydrogel to develop a formulation suitable for topical delivery. This study claimed that the combination of curcumin and imiquimod in the nanoemulgel system to prevent the appearance of psoriasis-like symptoms compared with the imiquimod nanoemulgel and imiquimod gel formulation alone.

Researchers have shown that capsaicin can inhibit the growth of some important food-borne pathogenic microorganisms, such as *Listeria monocytogenes*, *Helicobacter pylori*, *Pseudomonas aeruginosa*, *Botrytis cinerea*, *Aspergillus niger*, *Staphylococcus aureus* and *Penicillium expansum*. It has also been shown to exhibit anti-virulence activity against *Vibrio cholerae*, and *Poephyromonas gingivalis*. Capsaicin is the main capsaicinoid playing its role as antimicrobial and antifungal agent and help as an analgesic in anti-inflammatory diseases like rheumatism, arthritis and diabetic neuropathy. A pharmaceutical carbopol-based formulation containing capsaicin extracted from *Capsicum annuum* fruits, was synthesized by Goci et al. [10], and its in vitro release, antimicrobial and antifungal properties were evaluated against some bacterial and fungal strains.

Moving ahead, breast cancer is the most prevalent cancer in women around the world. According to U.S. Breast Cancer statistics [11], one out of every eight women will suffer breast cancer at certain times of her life. Treatment options may differ on the basis of stage of the disease, characteristics of the patient, and general health status. Treatment methods such as surgery, radiation therapy, hormone therapy, chemotherapy, targeted therapy, or herbal treatments are gaining popularity and are continuing to develop rapidly. Recently, researchers are investigating biochemical and therapeutic routes that can utilize natural anticancer agents/extracts obtained from plants, microorganisms, and animal sources. In an article in this Special Issue, the anticancer activity of an extract isolated from a medicinal plant *Dorycnium pentaphyllum* subsp. *haussknechtii* was evaluated against different breast cancer cell lines, to determine invasion, adhesion, and lipid peroxidation [12]. The extract showed time- and concentration-dependent cytotoxic effects on MCF-7 breast cancer and MCF-12A as the non-cancerous cell line, in a XTT assay. The optimum concentration of the extract was ascertained based on apoptotic activity, invasion, adhesion, and lipid peroxidation assays. Bioinformatic analysis revealed that the extracts to contain uercetin (an antioxidant compound). The mechanism of action underlying was elucidated along with the observed anti-proliferative effects, which caused tumoral cell death, but without any cytotoxic effects on human healthy breast cells. The authors proposed this to the high affinity of quercetin towards the enzymes: proto-oncogene serine/threonine-protein kinase (PIM-1) and hematopoietic cell kinase (HCK), which are involved in breast cancer.

Liposarcoma (LS) is associated with considerable morbidity and mortality and particularly poor prognosis, due to local recurrence and tendency to metastasize to the lungs and liver. Despite the development and clinical utilization of new targeted chemotherapeutic agents, improved radiation targeting, and surgical techniques, only a small increase in overall survival of sarcoma patients’ have been demonstrated over the last two decades. Hence, there is a marked need to explore much more effective and nontoxic antineoplastic drugs to enhance locoregional disease control and overall survival in sarcoma patients. A natural bioactive compound, 6-Shogaol, has been shown to exhibit anticancer properties in certain types of cancer. The study by Yadav and Jang [13] investigated the anticancer properties of 6-Shogaol against two different human liposarcoma cell lines (SW872 and 93T449 cells). This study revealed that 6-Shogaol to inhibit the growth of both cell types without affecting the normal cell lines (normal 3T3-L1 preadipocytes). The potential anticancer mechanisms of 6-Shogaol were attributed to the nuclear DNA fragmentation, increased sub G1 population, activation of the intrinsic caspase pathway, and PARP cleavage. In addition, 6-Shogaol also upregulated the expression and phosphorylation of GRP-78, eIF-2α, ATF4, and CHOP, which are known ER stress markers in SW872 cells, illustrating the induction of ER stress. The findings illustrated that 6-Shogaol to have strong antigrowth and pro-apoptotic effects on SW872 cells by regulating the intrinsic caspase pathway, oxidative stress, STAT-3, AMPK, and ER stress.

Nanotechnology is being explored to create nutraceutical formulations with improved efficacy. Stănciuc et al. [14], crosslinked β-lactoglobulin (β-LG) with caffeic acid (CA) using transglutaminase (Tgase). This study provided knowledge about the binding affinity and interaction mechanism of native and cross-linked proteins at different temperatures (25 to 95 °C). The authors claimed that β-lactoglobulin and CA were bound to each other through weak Van der Waals’ forces and hydrogen bonding. They also confirmed that there were various CA binding sites on the surface of heat-treated and partially denatured β-LG proteins. The authors reported that CA-β-LG and CA-β-LG_Tgase_ complexes (ratio 1:1) to have exhibited significantly higher antioxidant activity and inhibitory effects on α-glucosidase, α-amylase, and pancreatic lipase, which are the enzymes associated with metabolic syndrome, when compared to the native proteins.

In another study, Grigore-Gurgu et al. [15] used microencapsulation to enhance the biological properties of two bioactive components (flavonoids and peptides) in a single formulation. They microencapsulated flavonoids extracted from yellow onion skin using papain-hydrolysed peptides extracted from bone tissue from phytophagous carp (*Hypophthalmichthys molitrix*). The peptides contained some specific binding sites for flavonoids and revealed the involvement of hydrogen bonds and weak van der Waal interactions in microcapsule formulation. These hydrolyzed proteins improved the encapsulation efficiency of flavonoids. The microencapsulated samples exhibited no sign of cytotoxicity in a cell culture model.

Polysaccharide microgels loaded with ellagic acid were developed by Alhakamy et al. [16] to enhance its controlled release in the alimentary tract to treat peptic ulcers. The ellagic acid-loaded microgels, which were formed from calcium pectinate, were shown to significantly improve the gastric ulcer index in rats when compared with native ellagic acid.

Overall, this Special Issue has demonstrated the potential of a broad range of bioactive molecules isolated from plant-based materials to improve human health and well-being, as well as the potentiality for nanotechnology to improve the efficacy of these bioactive molecules. Clearly, this is an important area and there is need for much more research to be undertaken in the future to identify other kinds of bioactive molecules and nanoparticle-based delivery systems and prove their efficacy.

## Figures and Tables

**Figure 1:**
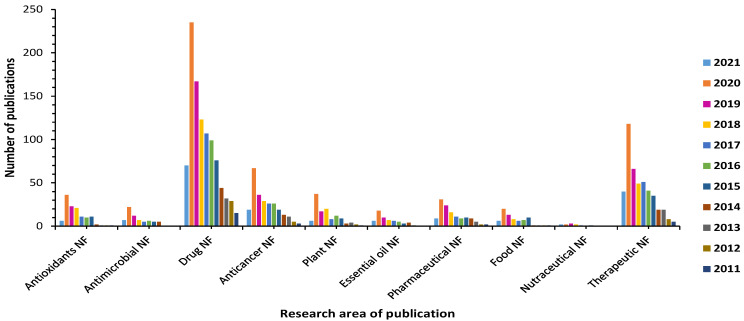
Graphical representation of number of publications from Web of Science in the field of antimicrobial, antioxidants, drug, anticancer, plant, essential oil, pharmaceutical food, nutraceutical and therapeutic nanoformulations from January 2011 to May 2021. NF = nanoformulations.

## Data Availability

Not applicable.
